# Building the genomic nation: ‘Homo Brasilis’ and the ‘Genoma Mexicano’ in comparative cultural perspective

**DOI:** 10.1177/0306312715611262

**Published:** 2015-12

**Authors:** Michael Kent, Vivette García-Deister, Carlos López-Beltrán, Ricardo Ventura Santos, Ernesto Schwartz-Marín, Peter Wade

**Affiliations:** Social Anthropology, University of Manchester, Manchester, UK; Facultad de Ciencias, Universidad Nacional Autónoma de México, Mexico City, Mexico; Instituto de Investigaciones Filosóficas, Universidad Nacional Autónoma de México, Mexico City, Mexico; Fundacão Oswaldo Cruz – Escola Nacional de Saúde Pública, Rio de Janeiro, Brazil; Department of Anthropology, Durham University, Durham, UK; Social Anthropology, University of Manchester, Manchester, UK

**Keywords:** biomedicine, Brazil, genetics, health, Latin America, Mexico, nationalism, race

## Abstract

This article explores the relationship between genetic research, nationalism and the construction of collective social identities in Latin America. It makes a comparative analysis of two research projects – the ‘Genoma Mexicano’ and the ‘Homo Brasilis’ – both of which sought to establish national and genetic profiles. Both have reproduced and strengthened the idea of their respective nations of focus, incorporating biological elements into debates on social identities. Also, both have placed the unifying figure of the mestizo/*mestiço* at the heart of national identity constructions, and in so doing have displaced alternative identity categories, such as those based on race. However, having been developed in different national contexts, these projects have had distinct scientific and social trajectories: in Mexico, the genomic mestizo is mobilized mainly in relation to health, while in Brazil the key arena is that of race. We show the importance of the nation as a frame for mobilizing genetic data in public policy debates, and demonstrate how race comes in and out of focus in different Latin American national contexts of genomic research, while never completely disappearing.

## Introduction

On the 11 May 2009, during a dramatic and highly mediatized official ceremony, Mexican president Felipe Calderón received what was publicly called the ‘Genome of the Mexican mestizo’ from the hands of Gerardo Jiménez-Sánchez, the director of the Mexican Institute of Genomic Medicine (INMEGEN). Jiménez-Sánchez presented the gift as ‘the book of life … for Mexicans, by Mexicans, of Mexicans’. In his speech, president Calderón described the ‘map of the Mexican genome’ as a vital instrument for understanding, preventing and treating serious diseases that affect the Mexican population. The symbolism and language of the presentation conjured issues of national identity: the president was enacting metaphorical ownership of biological clues to the identity of ‘*el mexicano*’, the product of centuries of genetic admixture, giving substantive content to the abstract notion of ‘genomic sovereignty’ (Seguín et al., 2008).

In April 2000, amid the celebrations of the 500th anniversary of the Portuguese arrival in Brazil, geneticist Sérgio Pena et al. (2000) published an article, ‘Molecular portrait of Brazil’, in the popular science magazine *Ciência Hoje*. Pena and colleagues emphasized the mixed genetic ancestry of self-identified white Brazilians, as evidence of the mixed nature the Brazilian population in general. They presented genetic knowledge as a potential antidote to racism, which had become seen as one of the main social problems of the country:

It might be naïve on our part, but if the many white Brazilians who have Amerindian and African mitochondrial DNA became aware of this, they would better value the exuberant genetic diversity of our population, and, who knows, they might construct a more just and harmonious society in the twenty-first century. (Pena et al., 2000: 25)

Later, Pena (2002) coined the term ‘Homo Brasilis’ to refer to the specificity of the Brazilian population, resulting from a ‘unique … process of genetic mixture’ (p. v).

Mexico and Brazil were, in the first decade of the 21st century, well situated, economically and scientifically, to profit from the biotechnological possibilities enabled by the race for the human genome. In both countries, the context for national genomic projects included access to high-tech sequencing and bioinformatics, and well-trained scientists who were already participating in cutting-edge research. In both places, projects analysed similar scenarios, using the conceptual and technical tools of population genetics and the guiding frames of questions about genetic admixture. These projects addressed the past, present and future of the nation, placing the figure of the mestizo/*mestiço* (mixed person) at the centre of the nation. However, while both research endeavours hinted at solutions to some of their countries’ most crucial problems, such as health, sovereignty and national integration, each privileged local issues: concerns about health took centre stage in Mexico, while dilemmas about how to confront racism and racial inequalities were paramount in Brazil.

There were other fundamental differences between the two endeavours. What has generally been referred to as ‘the Genoma Mexicano project’ (henceforth GM) was a large-scale, coordinated, centralized and government-led endeavour, which sought to address nation-wide biomedical priorities, which were linked to rocketing rates of obesity and diabetes. The GM mobilized an extant stable social mestizo identity and redrew it in population genomic idiom, preserving its basic ready-made outline; race was implicit in the figure of the mestizo, but racism was not a central concern (Hartigan, 2013; López-Beltrán and Vergara Silva, 2011). In contrast, research on Homo Brasilis (henceforth HB) emerged from the scientific field in a decentralized way, was centred on Pena as one of Brazil’s best-known and most media-savvy geneticists, and became incorporated into debates on public policy and national identity – especially ones about the state’s controversial race-based affirmative action initiatives. Pena and others used genetic data to deconstruct existing racial identities, and to lobby for a raceless nation.

Both GM and HB reproduce the nation by generating national genetic profiles, deploy biology in discourses on national identity and locate (or relocate) the figure of the mestizo at the heart of such identity. But, given their different national socio-political contexts, GM and HB have distinct trajectories, which have established different kinds of relationships between genetics and social identity. This article analyses the convergences and differences of GM and HB.^1^

This analysis addresses the complex ways in which genetic research becomes articulated with socially imagined identities and nations. Since the 1990s, the development of new molecular techniques has enabled direct biological connections between contemporary populations and (a proportion of) their ancestors, as well as the production of genetic profiles that indicate degrees of mixture and the relative contribution of ancestral populations (Amerindian, African and European in the case of Latin America). Both geneticists and non-scientists have at times used genetic knowledge as an element in their interpretations of social groups, their histories and their futures (e.g. Wailoo et al., 2012). In this article, we focus primarily on geneticists’ discourses about individual and collective identities, and the forms of ‘biosociality’ (Rabinow, 1996) and ‘imagined genetic communities’ (Simpson, 2000) that these discourses imply, in terms of possible collective identifications that are based on presumed genetic characteristics (Brodwin, 2002; Kent, 2013; Nelson, 2008; Pálsson, 2007).

Academic debates concerned with the relationship between genetics and identity – particularly those in the United States, but also those in Europe – have often focused on race and ethnicity. Some social scientists have emphasized the role of genetics in reproducing racial categories, albeit in altered form (Fujimura et al., 2008; Fullwiley, 2008; Koenig et al., 2008; Montoya, 2007; M’charek, 2013; Palmié, 2007). Others have raised concerns about the divide between notions of genetic ancestry, on the one hand, and indigenous conceptions of identity, origins and belonging, on the other (Reardon, 2005; TallBear, 2007). With some exceptions (M’charek et al., 2014b; Nash, 2013; Pálsson and Rabinow, 1999; Wailoo et al., 2012), the relationship between genetics and imagined national identities has received less attention, in spite of considerable research on genetic sovereignty, national databases and the nation as a collective of biological citizens with different levels of inclusion (Benjamin, 2009; Heath et al., 2004; Hinterberger, 2012a; Pálsson, 2007; Rabinow, 1999; Rose and Novas, 2005; Schwartz-Marín and Silva-Zolezzi, 2010). Yet in Latin America, the nation – as well as the mestizo that stands at the heart of national identity constructions – has figured as much or even more in human genetic research than has race or ethnicity (Hartigan, 2013; Santos and Maio, 2004). In Latin American countries, in particular in Brazil and Mexico, mixture is granted higher levels of interpretative priority in understanding the national population than racial categories, although the overtness of race varies widely within Latin America – for example, between Brazil and Mexico (Wade et al., 2014b). The figure of the mestizo allows for an ‘absent presence’ of race – in which race appears to be absent, but is present in the traces it has made (M’charek et al., 2014a) – insofar as there is a racialized structure embedded or hidden in the mestizo, but the nation occupies centre stage (Wade et al., 2014a). Brazil and Mexico offer a privileged context for exploring the relation between genetics, national identities and nation-building efforts, in part because of the powerful articulations between biological and social repertoires established by the mestizo as a long-standing scientific and social object.

While arguing for the specificity of Latin America, this article qualifies the homogeneity of race and identity often projected onto the region. Although the GM and HB projects are similar in many respects, their trajectories and the public projections of their results have been quite different. Therefore, it is necessary to guard against overly broad regional generalizations, and avoid juxtaposing Latin America as a block with the US context.^2^ The sections of this article cover: (1) the historical background of relationships between biology and nation in both Mexico and Brazil, (2) the context of genetic research for each country, (3) the characteristics of the GM and HB projects, and (4) the convergences and divergences between the two projects, in terms of how they construct the nation and relate to public policy.

## Historical context

In Brazil and Mexico, there is a long tradition of thinking about the nation through its biological constitution (Stepan, 1991). In the late 19th and the early 20th centuries, physical anthropology played a central role in debates about national identity in both countries (García Murcia, 2013; Santos, 2012; Vergara Silva, 2013), analysing the history and constitution of national populations, and addressing the desirable future of the nation. In Brazil and Mexico, life scientists, social scientists and other intellectuals perceived their nations as racially heterogeneous and interpreted race-based differences as the natural bases of social hierarchies. Additionally, European and North American racialist theories influenced many (though not all) of these thinkers to interpret admixture as a form of degeneration, which damaged the fitness and productive potential of the nations’ populations. Close connections were established between the health of the nation’s social body and the health and vitality of citizens’ individual bodies (Appelbaum et al., 2003). In Brazil, ‘whitening’ was understood as a means of improving the nation, and state policies encouraged the immigration of approximately six million Europeans. Mexico had less immigration, but still ‘racial homogenization was an imperative on which everyone in nineteenth-century Mexico agreed’, even if ‘the type of citizen required by the new nation and the formula for creating such a citizen were a source of disagreement’ (López-Beltrán and García-Deister, 2013: 393).

The period from 1910 to 1930 saw the emergence of nationalist projects in Brazil and Mexico, with some resistance to theories that condemned racial mixture. Physical anthropologists, along with other intellectuals, re-interpreted mixture in positive terms, and discourses that emphasized common features and harmony became central to debates on national identity. In these discussions, mixture was conceived in terms of history and culture, as well as in terms of biology. Academics and intellectuals who engaged in nation-building efforts reflected on the national character, by systematically describing the physical and psychological peculiarities of the Mexican and Brazilian people – their forms of life, gestures, temperament and history. From the 1930s, the hybrid figure of the mestizo (in Mexico) and the mestiço (in Brazil) became central to the construction of national identity.

Mexico’s solution to racial heterogeneity involved protecting indigenous peoples and simultaneously incorporating them into the mestizo body of the nation. This assimilationist project, which was known as *indigenismo*, was facilitated by biological crossing and educational acculturation. Physical anthropology, which emerged as the key scientific discipline for forging national identity (García Murcia, 2013; Rutsch, 2001), was instrumental. The project was successful insofar as the brown mestizo – descendant of a mythified original encounter between Spanish male and female *indígenas –* came to be synonymous with the typical Mexican (Gómez Izquierdo and Sánchez Díaz, 2011). Influenced by the work of philosopher and politician José Vasconcelos (1997 [1925]) author of *La raza cósmica*, a group in the National Autonomous University of Mexico, under the name Hiperión, undertook the task of ‘making manifest the phenomena constituting what it meant *to be* Mexican, and ultimately, with *prescribing* a “mode of being” appropriate for the creation of an authentic and, consequently, a responsible *homo mexicanus*’ (Sanchez, 2008: 442).

Over a similar period, the idea of the *raça brasileira* was debated by Brazilian anthropologist Roquette-Pinto and sociologist Oliveira Vianna, while a Brazilian *moreno* (brown) ‘meta-race’ was discussed, somewhat later, by Gilberto Freyre (Hofbauer, 2006; Lima and Sá, 2008; Oliveira Vianna, 1923). In Brazil, Freyre’s work has been influential, with its argument that the intense and relatively consensual mixing of white Europeans, black slaves and Indians – *mestiçagem* – blurred differences so much that, nowadays, there are no clear-cut distinctions between them, only a racial continuum from the whitest to the blackest individual (Freyre, 1936). This point has often been pushed further to argue that Brazil has become a ‘racial democracy’, in which relations between people of different colours are relatively egalitarian and racism plays a minor role. This view became official ideology and was actively promoted as part of the nationalist policies of the Getúlio Vargas administration (1930–1945), and during the military dictatorship (1964–1985). In the 1950s, United Nations Educational, Scientific and Cultural Organization (UNESCO) sponsored studies of Brazil as a possible example of successful race relations, with mixed results (Maio, 2001; Wade, 2010: 52–59).

Interpretations of the nation that focuses on unity and the mestizo have maintained different levels of legitimacy in the two countries into the present. Since the 1990s, there have been political shifts all over Latin America towards ‘multiculturalism’, involving the legislative recognition of indigenous and, to a much lesser extent, Afro-descendant groups. These shifts have ranged from the recognition of land-title claims to affirmative action policies (Sieder, 2002). In Brazil, after the UNESCO studies, social scientists and black movements highlighted the continued existence of profound racial inequalities, and presented Brazilian society as consisting of economically and socially differentiated white and black segments (Guimarães, 1999; Telles, 2004). These perspectives were the basis for multiculturalist policies focused on Brazil’s black population, including racial quotas for public employment and university access (discussed in this special issue in Kent and Wade, 2015), and race-based health policies. Such policies have, in turn, been strongly challenged by some academics, the mass media and (mostly centre-right) political parties, who have proposed universal or socio-economic criteria for inclusion. With a renewed focus on the process of mixture, this critique has centred on the argument that, in Brazil, inequalities are more class-based than race-based (Fry, 2005; Fry et al., 2007; Maio and Santos, 2005).

During the post-Revolutionary period (1940–2000) in Mexico, a ‘mestizo ideology’ (Gómez Izquierdo and Sánchez Díaz, 2011) emerged through successful educational and propaganda programmes devised by nationalist intellectuals associated with the ruling elite. Fuelled by the regime of the Partido Revolucionario Institucional (PRI), this ideology ‘acted as a mask to the transcendent power of [white] social elites’ (Gledhill, 2002: 39), reinforcing a pigmentocratic social system behind a false impression of homogeneity and equality (Telles and Project on Ethnicity and Race in Latin America, 2014). The figure of the mestizo was articulated around public policies that excluded those – indigenous groups (Navarrete Linares, 2009), Afro-descendants (Cunin and Hoffmann, 2013) and immigrant populations (e.g. Chinese, Jews) – considered to be incompatible with the national project of *mestizaje* (mixture) (Gleizer, 2011; Gómez Izquierdo, 1992).

More recently, there has been growing critical inquiry into the biological and cultural aspects of the mestizo, much of which treats overemphasis on the mestizo as an obstacle to the appreciation of diversity in Mexico (Gall, 2007; Tenorio, 2006; Viqueira, 2010). Multiculturalist reform, focusing on indigenous land rights and political autonomy, has been a goal of indigenous and other social movements, such as the Zapatistas, and there has been some shift towards state multiculturalism. This is evident in official, albeit vague, recognition of indigenous cultural practices, implementation of some ‘intercultural’ education programmes and attribution of some political autonomy to indigenous communities (De la Peña, 2006). But the multiculturalist shift has been less marked in Mexico than in Brazil, and the former has not resulted in affirmative action policies based on race or ethnicity.

## The genetic field in Brazil and Mexico

In Brazil, human population genetics is a well-established academic field that has been growing steadily since it emerged in the late 1950s (Souza and Santos, 2014). Early on, numerous studies with ‘classical’ genetic markers were conducted at a number of universities to understand the formation and evolution of the Brazilian population from a genetic perspective.^3^ These ‘racial mixture studies’ aimed to establish the relative contributions of ‘white/Caucasian’, ‘black/Negroid’ and ‘Indian’ populations to the gene pool of Brazilian populations. Key authors – such as Francisco Salzano, Pedro Saldanha and Newton Freire-Maia – highlighted the importance of the genetic diversity of the Brazilian population for understanding of admixture and inter-ethnic relations on a global scale (e.g. Salzano and Freire-Maia, 1970). To them, the Brazilian population was unique, as a result of the biological processes that had given rise to it. These researchers had strong international ties, published mostly in English, and established a permanent dialogue with the global genetic field. In addition to doing human population genetic research, many of them were active in medical genetics (Souza and Santos, 2014).

The 1990s saw a methodological shift towards analyses of molecular DNA. With this came a rapid expansion of the scale of genetic ancestry research in Brazil. The central concerns of such research, however, were largely the same: mixture, the biological diversity of the Brazilian population and the relative contribution of its founding populations – now redefined in geographical terms, such as African, Amerindian and European – to its contemporary gene pool. At present, there are several research centres that focus particularly on these issues, including the Federal University of Minas Gerais in Belo Horizonte, where Pena is located. Thus, when Pena started his studies on what became HB, he drew on a well-established sub-discipline.

In Mexico, medical genetics was the first branch of human population genetics to be developed (Barahona, 2010). Although there were attempts to assess human diversity and mestizaje using genetic techniques in the 1920s and 1930s (Saade Granados, 2009), it was not until the 1950s that medical geneticists educated abroad – most notably Rubén Lisker – set out to study genetic variation of blood (i.e. the distribution of blood groups and abnormal haemoglobins) in Mexican populations (Suárez-Díaz, 2014; Suárez-Díaz and Barahona, 2013). Although these studies were linked to biomedical projects aimed at assessing genetic conditions of interest to public health, the topic of levels of admixture in different subpopulations was a common denominator. Like Mexican physical and cultural anthropology, these studies initially focused on indigenous populations, and later turned to evaluate the molecular effects of admixture in ‘Mexican mestizo populations’ (López-Beltrán et al., 2014; López-Beltrán and García-Deister, 2013).

The arrival of DNA sequencing technologies at the turn of the 21st century allowed more refined estimates of the degree of mestizaje (understood as proportions of European, Amerindian and African genetic ancestry). In contrast to Brazil, where research like Pena’s was decentralized and carried out by individuals and small teams, in Mexico in the early 2000s, a group of politically influential physicians set out to organize the dispersed population genetics endeavours, to define the strategy that Mexico would follow in order to take advantage of the newly generated tools and knowledge. This led to the creation of the National Institute for Genomic Medicine (INMEGEN) in July 2004. INMEGEN’s medically oriented Mexican Genome Diversity Project (MGDP) was, like biomedical projects of the previous century, accompanied by anthropo-historical questions. The GM, which encompassed this project and its resulting ‘Map of the Mexican Genome’, aspired to become both a biomedical research tool for the genetic basis of diseases specific to the Mexican population, and a molecular portrait of the Mexican mestizo (see, in the current issue, García-Deister and López-Beltrán, 2015).

## The Homo Brasilis

The ‘Molecular Portrait of Brazil’ analysed the genetic ancestry of self-identified white men from different regions of the country. It revealed that, while their paternal ancestry was almost exclusively European, their maternal lineages had ‘surprisingly high’ proportions of Amerindian (33%) and African (28%) ancestry. Since then, working with different sets of collaborators from various universities in Brazil, Pena has produced a number of further studies on the genetic ancestry of the Brazilian population.^4^ Drawing on a highly influential tradition of genetic research (Cavalli-Sforza et al., 1994; Lewontin, 1972), Pena has systematically argued that there is no biological basis for the idea of race. On his account, it is impossible to differentiate between racially defined groups within the Brazilian population at the genetic level. >From this body of research, an image of the Brazilian population that is unified and mestiço emerges. Sometimes, the image is implicit, such as when Pena addresses questions of pharmacogenomics and biomedicine (Suarez-Kurtz et al., 2007). At other times, the image is explicit: when Pena’s focus is on the genetic ancestry of Brazilians (Pena et al., 2009, 2000, 2011); when he engages with issues of public policy, such as affirmative action (Pena and Bortolini, 2004); or when he discusses differential health policies aimed at Brazil’s black population (Pena, 2005).

In 2002, Pena published an edited book on the Brazilian population, which featured contributions by anthropologists, linguists and historians, in addition to several chapters on genetics. He justified the term HB, in the title, as follows: ‘[in Brazil] a process of genetic mixture was initiated that is unique in the entire history of Humanity, generating the contemporary Brazilian, which we decided to call, a little irreverently, Homo Brasilis’ (Pena, 2002: v). Although this term has not resurfaced in subsequent research, we have decided to use it in this article, to reflect the research object at the heart of Pena’s ancestry studies: the particular Brazilian population that is the result of unique genetic processes, a research object previous geneticists had already described (Souza and Santos, 2014).

Research published in 2003 analysed the autosomic DNA – the recombinant part of the genome that is particularly suited to revealing levels of admixture – of samples collected among individuals classified according to the main census categories of *preto, pardo* and *branco* (black, brown and white). Results revealed that while variation in genetic ancestry between individuals within each category was considerable, such variation between categories taken as a whole was relatively small. The authors concluded that, in Brazil, there is only a weak correlation between people’s physical appearance (their colour or race, in Brazilian terms) and their genetic ancestry (Parra et al., 2003: 177). On the basis of such data, Pena has argued repeatedly that ‘the only way of dealing scientifically with the genetic variability of Brazilians is individually, as singular and unique human beings in their mosaic genomes and in their life histories’ (Pena and Birchal, 2006: 19). According to Pena, Brazilians should be classified simultaneously as an undifferentiated mestiço population – the HB – and as a collection of individuals that are ‘equally different’ (Pena, 2009).

Since 2006, Pena’s research has argued for dissociation between physical appearance and genomic ancestry in Brazil. He has placed particular emphasis on the admixed character of Brazil’s black population, highlighting the predominance of European ancestry among individuals self-classified as pardo (80%), as well as the lower than expected proportions of African ancestry (40%–50%) among those categorized as preto (Pena et al., 2009, 2011). Finally, while the ‘molecular portrait’ revealed significant regional differentiation within Brazil in terms of genetic ancestry (Pena et al., 2000), Pena’s most recent research has focused on deconstructing such differences. Using samples collected in four of the country’s five macro-regions, it led to the conclusion that ‘the genomic ancestry of individuals from different geographical regions of Brazil is more uniform than expected’ (Pena et al., 2011: 1). Pena’s research has generated an image of the Brazilian population that is unified and mestiço, subsuming underlying differences in terms of both race and region – the two main sources of differentiated social identities in Brazil.

## The Genoma Mexicano

In 1999, Gerardo Jiménez-Sánchez (a medical doctor with a PhD in human genetics from Johns Hopkins), supported by his senior colleague Guillermo Soberón Acevedo, whose career included terms as Mexican Secretary of Health (1982–1988) and rector of Mexico’s National University (UNAM), began to propose a national research programme on medical genomics. From the start, and in contrast to comparable projects in Brazil, the initiative was linked to mainstream state institutions. Lobbying efforts for the creation of INMEGEN began under the aegis of the Fundación Mexicana para la Salud (FUNSALUD), a politically powerful institution that links private and public interests around health-related issues, then headed by Soberón. Initial meetings brought together further potential funders: the Mexican Ministry of Health, the National Autonomous University (UNAM) and the Mexican Council of Science and Technology (CONACYT, Mexico’s main state funding agency for scientific research).

The first results of the Human Genome Project in 2000 indicated to Jiménez-Sánchez and Soberón that Mexico could not afford to be outside the genomic revolution, and needed to create a genomic research institute. A 2001 Feasibility Study (IFS) helped potential investors to foster informal support for the development of INMEGEN, and became the platform for circulating the promises of genomic medicine to strategic audiences, including members of Congress.

Soon after, the Consortium for the Creation of the Institute for Genomic Medicine was created to promote the project, with the backing of eminent physicians such as Juan Ramón de la Fuente (former Minister of Health and at the time Dean at UNAM) and Julio Frenk (Minister of Health at that time, and current Dean of Harvard’s School of Public Health), and corporate figures such as pharmaceutical tycoon Antonio López de Silanes. Due to his experience and networks, Jiménez-Sánchez was chosen to lead an initiative that INMEGEN lobbyists deliberately compared with the Human Genome Project. From July 2004, when INMEGEN was created, to November 2010, when he stepped down as director, Jiménez-Sánchez was the face of Mexican genomic medicine. The task was to bring Mexican medical genomic research to a global level of excellence.

Under Jiménez-Sánchez, the MGDP aimed to be the catalyst of this change, by attempting to describe the genetic heterogeneity and the specific genetic characteristics of the Mexican population, and exploring haplotype–disease associations (Jiménez-Sanchez et al., 2001). Once the sampling and genotyping was achieved, besides the construction of the haplotype platform for supporting further biomedical research with Mexican patients, a series of genetic analyses of population admixture were performed to measure genetic heterogeneity, heterozygosity, genetic distances from other continental populations and between Mexican regions, and proportions of European, Amerindian and African genetic ancestries. During the project, samples from a Zapotec indigenous group were incorporated into the analysis, to serve as a reference point for assessing Amerindian genetic ancestry (López-Beltrán et al., 2014; Silva-Zolezzi et al., 2009; Taylor-Alexander and Schwartz-Marín, 2013).

Although the project set out to measure diversity, the mestizo, as an object recognizable by both political and scientific communities, remained a dominant figure: ‘The initial results of the MGDP published in 2009 show that although some regional genetic differences exist between Mexican subpopulations, these are similar enough to be considered as a single group’. Scientists further concluded that, despite considering Mexicans a relatively homogeneous national population (reinforcing the well-known saying that ‘we are all mestizos’), there were some ‘genetic differences between mestizos of some regions of Mexico’, mainly due to ‘differences in ancestral contributions by European and Amerindian groups’ (Jiménez-Sánchez et al., 2012: 1186–7). Although African ancestral contributions appeared in varying percentages in the samples, they were not enough to question the well-established nationalist narrative that the Mexican mestizo is the outcome of European and Native American admixture.

During all the phases of the MGDP, INMEGEN devoted resources to public engagement activities: conferences, media coverage, museum exhibits, publications (including comic books and public science articles). Three discursive elements were central to these efforts: (a) the idea that the Mexican population has distinctive genetic peculiarities; (b) the idea that such peculiarities derive from the events of admixture starting 500 years ago; and (c) the idea that the distinctive genetic make-up of Mexicans can be represented as a ‘Map of the Mexican Genome’. Few critical reactions were triggered by these campaigns. INMEGEN succeeded in mobilizing a nationalist rhetoric that was already in place in order to bolster the concept of a ‘GM’. The genetic portrayal of Mexicans as bodies assembled from portions of European, Amerindian and (to a lesser extent) African ancestry was not news. INMEGEN’s research translated the notion of national mestizo identity, a concept already underwritten by older genetic studies, such as those by Lisker (Suárez-Díaz, 2014), into the now more publicly disseminated language of genetic ancestry. INMEGEN underscored the fact that Amerindian ancestry contributed distinctive qualities to Mexican mestizaje, which echoed the perception that a pre-Hispanic past also makes contemporary Mexicans culturally unique.

## The construction of national research objects and essentialized mestizo nations

As simultaneously scientific and socio-political projects, the GM and HB each established more pronounced exchanges between biological and social repertoires than is usually the case in genetic research. Both projects focussed on nations and their mixtures, placing figurative genomic mirrors in front of national populations, and calling on members to recognize their ‘real’ characters. In this way, both the GM and HB framed relationships between genetic ancestry and social identity in ways that essentialized conceptions of the nation and the mestizo.

Pena’s research relies on constructs such as Brazil and its population, HB and the Molecular Portrait of Brazil, and the nation as a discrete unit of sampling and analysis. In his publications, Pena has systematically used his genetic perspective to reject alternative categories, particularly those based on race, and, more recently, region. Notably, Pena (2008) dissociates ancestry from phenotypic appearances, in order to ‘uninvent’ standard Brazilian conceptions of race. Such conceptual work has produced a generic, yet individualized mestiço – the sum of Brazil’s 190 million ‘equally different’ individuals – who transcends underlying differences and defies racial categorization. However, neither the Brazilian nation as an overarching unit, nor the mestiço as its key element, has been questioned in Pena’s research: HB replaces reified racial categories with essentialist conceptions of the nation and the mestiço.

According to Pena (2008), the world’s population consists of six billion equally different individuals and Brazil’s 190 million individuals must be seen likewise. Yet, Brazil’s population is also seen as *singular*. Pena has attempted international dialogue, using the Brazilian population in order to think about universal issues, such as race relations, and to explore the lessons that Brazil has to teach to the world (Pena, 2008, 2009; Suarez-Kurtz et al., 2007). An example is his ‘We R No Race’ campaign, which aimed to map the genetic ancestry of participants in the 2014 World Cup of football, held in Brazil.^5^ Pena has also conceptualized the Brazilian population as a synthesis of the world’s population – the mixture of Asian/Amerindian, European and African roots. Thus, in Pena’s approach, the Brazilian population represents a singularity that is also universal at the same time.

Social appropriations of Pena’s research deploy more explicitly essentialist ideas of a genetically unified mestiço population, and tend to appeal to the authority of science. Media features frequently refer to genetics in talk about ‘*the* Brazilian population’, or in affirmations that ‘we are all mestiços’. According to the 2008 ‘anti-quota manifesto’, which challenges the legitimacy of affirmative action policies that create racial quotas for university admissions (see below), ‘[t]he perception of mixture, which profoundly permeates Brazilians, in a way reflects realities proven by genetic studies’ (Daher et al., 2008). The *Globo* (2011) newspaper referred to Pena’s research to claim that ‘science has proven the nonexistence of the Afro-Brazilian’. While it has been used to deconstruct essentialized racial identities, genetic research in Brazil has also produced and widely disseminated essentialized notions of the nation and the mestiço. This suggests that, in the Brazilian case, the attraction of genetics lies in its potential to both consolidate and undermine identity constructions, to reify and to deconstruct, and to root such conceptual work in biological foundations.

Like HB, the GM constructed a national research object, based on an essentialist conception of mestizo. The GM depicted Mexico’s population as both a national and a singular entity: Mexico is now a mestizo nation and the typical citizen is a mestizo. This construction involved sampling the Zapotec people, who represented Amerindian genetic ancestry. Although they are Mexican citizens, and although their samples showed small degrees of genetic ancestral admixture (García-Deister, 2014), the Zapotec people were not considered ‘mestizos’. Instead, they were taken for granted as genetic representatives of the ‘indigenous’ category against which mestizoness is defined: the social identities of indigenous and mestizo were thus reproduced in a genomic idiom.

GM also took for granted the nation as its unit of analysis, reproducing Mexico’s national and internal boundaries in maps and charts. The quantifications of genetic ancestry produced by the GM are very similar to those produced by older technologies, following narratives of regional diversity that predict higher percentages of indigenous ancestry in the south and higher percentages of African ancestry in some coastal areas (Suárez-Díaz, 2014). The GM’s admixture figures thus appear like truisms, familiar and easy to accept.

In representations of genetics and the GM beyond the scientific field, the focus has been almost exclusively on Mexico, and on issues restricted to the national context.^6^ For example, the first results of the GM were reported in ways that affirmed the singularity of one genome. One newspaper article was headlined ‘Mexican genes: mixture of 35 races’: the article continued by saying that ‘the map of the human genome of Mexicans’ showed that ‘the genes of the Mexican population’ are ‘different from those of Europe, Asia and Africa’ and that ‘65% of the genetic make-up of Mexicans is unique and has been named “Amerindian”’ (Alcántara, 2007; López-Beltrán and Vergara Silva, 2011: 121). As we will show below, INMEGEN was founded amid discussions of ‘genomic sovereignty’ – Mexican control over Mexico’s genetic resources, seen as particular and as shaping the nation’s health in specific ways – which centred the research firmly within the national frame. In short, the case of the GM illustrates how circulation of genetic data in Mexico, like circulation of genetic data in Brazil, is grounded in conflations of socio-cultural identity and genetic ancestry.

While HB and the GM both constructed mestizo/mestiço nations, the two projects mobilized their ideas in different ways, reflecting differences in the scientific practices and concerns of Brazil and Mexico, respectively. First, while the Brazilian state has provided key sources of funding for Pena’s research, state support for genetic research in Brazil is not comparable to the Mexican state’s support for INMEGEN, a national research institute. Unlike Pena’s research, the GM visibly bore the stamp of its roots in central state commitments, including strategic alliances between the state and private foundations. Second, in Brazil, Pena and colleagues sampled from different regions of the country in a relatively ad hoc way, collaborating and sharing samples with other geneticists, and using different samples in various projects, according to availability. However, the collection of sample sets in Mexico was organized around a state-supported attempt to tap into the diversity of the nation: INMEGEN strategically created its own sample population, through a series of well-publicized *jornadas* (data-collection field trips) designed to recruit mestizo blood donors in three regions of the country: north, south and centre (García-Deister, 2014). Third, nationalist discourse plays only a background role in shaping HB. For example, foreign genetic approaches – those focussed on populations defined in ethnic-racial or geographic terms – are rejected as methodologically inappropriate for studying Brazilian admixtures (Pena, 2011; Suarez-Kurtz et al., 2007). In contrast, in Mexico, a discourse of genomic sovereignty framed the emergence of a powerful state-sponsored institution. According to this discourse, sufficient knowledge of the Mexican genome will benefit citizens, giving them future access to state-developed and regulated public health resources and policies, which will be tailored to the biological peculiarities of the population (Benjamin, 2009; Schwartz-Marín and Restrepo, 2013).

As we have shown, given the relative autonomy of Pena and his colleagues, on the one hand, and INMEGEN’s ties to the Mexican state, on the other hand, HB and the GM mobilized their respective conceptions of national mestizos differently. In the next section, we discuss distinctions between the two projects’ engagements with public policy debates about the nature and the future of the nation, health, and racial and ethnic diversity.

## Genetics, public policy and national identity

In both Brazil and Mexico, geneticists and interested political actors have actively sought to incorporate genetic knowledge into public policy debates. However, the politics of these pursuits in each country have been significantly different.

Pena disseminates his research, beyond the scientific field, through social science journals (Pena, 2005; Pena and Bortolini, 2004), popular scientific magazines and blogs (Pena et al., 2000; http://www.laboratoriogene.com.br/blog/), non-academic books (Pena, 2008, 2009) and frequent media appearances. He is involved in political debates about affirmative action policies aimed at Brazil’s black population, particularly debates around health and the heated debate on racial quotas for university access; the latter is discussed in Kent and Wade (2015), in this issue. The media,^7^ political parties (mostly centre-right) and some influential social scientists have also drawn on Pena’s studies to argue against race-focused, as opposed to class-focused policies (Fry, 2005; Fry et al., 2007; Magnoli, 2009; Maio and Santos, 2005; Santos et al., 2009). In addition to featuring prominently in the anti-quota manifesto (Daher et al., 2008), Pena’s research was key to the argument that racial quotas are unconstitutional (Kaufmann, 2009: 27–37; Kent and Wade, 2015): in 2010, the Democratas party presented the argument as a legal action to the Supreme Court, and Pena served as an expert witness.

Pena’s scientific research and political views are part of a wider Brazilian current that emphasizes pervasive mixture and a unified Brazilian identity. This competes with a post-1990 state multiculturalist approach to combatting racism and racial inequality, driven by the black social movement, academics and intellectuals and, importantly, the state. Pena frequently refers to authors associated with the racial democracy approach, such as Freyre and Darcy Ribeiro (Pena et al., 2000, 2009). The media draws on Pena’s work to affirm that ‘we are all mestiços’ and to criticize the idea of race-based groups as beneficiaries of affirmative action. For example, consider the case of the ‘Afro-Brazilian roots’ project, sponsored by BBC Brazil, which commissioned Pena to conduct genetic ancestry tests on nine black celebrities. The project was supposed to raise public interest in the partial African origins of the Brazilian population, but the results raised other concerns (see Kent and Wade, 2015). In this way, genetics served to re-centre national identity around the unifying figure of the mestiço, which had been partly displaced by post-1990 multicultural approaches that divided ‘white’ from ‘black’.

The debate on race-based public policies has been connected to notions of immanent crisis and the wellbeing of the nation. Critics of affirmative action warn that such policies might result in stronger racial divisions and even conflicts (Daher et al., 2008). Pena has conceptualized race as toxic, as a pathology that affects the health of the social fabric: ‘the survival of the idea of race is detrimental, as it is tied to the continued belief that human groups exist in a scale of value. This persistence is toxic, contaminating and weakening society as a whole’ (Pena, 2008: 6). The idea that genetics could be an antidote – not only for racism, but also for an apparent toxin of race in the national social imagination – has been politically influential. In Brazil, the debate into which genetic data have been drawn is no less than the future of the nation, in relation to long-standing debates about racial diversity and inequality.

In Mexico, however, genetic knowledge and the genetic mestizo have figured differently in issues of national policy and identity. As a lead INMEGEN researcher said of the GM, ‘The project is a study of diversity for biomedical applications; we think of it as a useful tool for linkage disequilibrium analysis and haplotype reconstruction’ (interview with García-Deister, 5 May 2010). Mexican scientists have been aligned firmly with the state’s approach to framing health priorities, especially the idea that obesity and diabetes are major problems in need of genetic solutions. Political campaigning by interested geneticists was a crucial aspect of the initial take-off of INMEGEN. Scientists and lobbyists justified investment of public money in INMEGEN, emphasizing the potential of genomic research to improve public health outcomes and reduce costs: genomic research promised to enhance genomic sovereignty, by tailoring future health provision to the Mexican citizens’ individual genetic profiles and reducing the nation’s dependence on foreign genomic research and pharmaceutical companies. In early 2009, INMEGEN scientists were conveniently on hand to offer a powerful scientific response to an unexpected outbreak of swine flu: on the brink of publication, INMEGEN’s Mexican Genome paper demonstrated that the national health system anticipated health hazards, such as the flu, and had cutting-edge genetics and local scientific talent to develop adequate responses. A newspaper report commented, ‘Only a story as strong as the decoding of the Mexican Genome could compete in Geneva, Switzerland, with the attention that the World Health Organization (WHO) had given to the health alert for AH1N1 flu’ (cited by Schwartz Marín, 2011: 241).

In Mexico, genetics is now intertwined with notions of crisis and the health of the nation. In the first decade of the 2000s, much of the country’s political discourse emphasized rising rates of obesity and diabetes as signals of a national medical crisis. The GM project was presented as a no-nonsense, radical, high-tech solution, one that could cut straight to genetic causes, located deep in the mestizo body: science and technology could help cure the ailing medical body of the nation (see García-Deister and López-Beltrán, 2015, in this issue). Thus, in Mexico, genomic research promised a better nation in the future (Schwartz Marín, 2011), and INMEGEN geneticists, the wider medical community and the state as whole agreed on what a better nation would look like – mestizo, of course, but a good deal leaner of body and generally more healthy. Critiques of INMEGEN did not focus on its overarching goals, but rather focussed on questions about whether projects were appropriately designed and cost-effective (López-Beltrán and Vergara Silva, 2011).

Despite initially being touted as a ‘race-based genome project’ (Guerrero Mothelet and Herrera, 2005; Hartigan, 2013), INMEGEN repeatedly denied race as a category. Nonetheless, in contrast with Brazilian geneticists like Pena, Mexican geneticists engaged with race in more implicit and less calculated ways. For example, their sampling and analysis indicates a clear divide between minority indigenous communities and the mestizo majority, and reproduces the *indio*/mestizo division that has long been central to the Mexican social imaginary. Meanwhile, the Mexican figure of the mestizo acts as a screen, hiding the racial underpinnings of typological categorizations in Mexico. The centrality of the mestizo to the GM has linked genetics and implicit racial concepts, rekindling the concept of race in the idiom of genetic ancestry. Researchers commonly use bio-geographical populations – Africans, Amerindians, Europeans – to represent genetic ancestries (López-Beltrán and García-Deister, 2013). Genetic markers are thus chosen in terms of bio-geographical ancestral lineages, but the markers appear to biologically differentiate whole populations (Fujimura and Rajagopalan, 2011; Hunt and Truesdell, 2013).

While the opacity of race dissociates Mexican mestizo bodies from racial markers (e.g. skin colour, facial features) associated with the past, the persistence of old ideas about *razas* is evident in contemporary Mexicans’ language. For example, press reports of the results of the GM said the state of Zacatecas was the ‘only sample with a half-indigenous, half-Spanish mestizaje’, while Sonora state ‘had the highest preponderance of European genes’ and Guerrero ‘the highest level of African genes’.^8^ The GM’s tacit relation between genetics and race reifies a crypto-racialized mestizo. Nonetheless, there is consensus in Mexico about the central issue of genomic research (viz. health), which avoids the kinds of divisive issues of race and multiculturalism that characterize genomic research in Brazil.

## Conclusion

We have illustrated how HB and the GM, two different genomic research projects on national populations, converged around concepts of mixture – mestizaje, *mestiçagem* – and the mestizo. In the early and middle parts of the 20th century in both Brazil and Mexico, the mestizo became central to self-conscious processes of state-supported ideological nation building. The two countries both have long-standing motivations to define themselves in ways that resist cultural dominance by the United States, and genomic research in both countries has thus attempted to develop genomic resources, techniques and knowledge that produce distinctive national profiles. Moreover, Mexico and Brazil both participate in a global scientific enterprise that primarily recognizes them as countries that can offer examples of admixed populations (Burchard et al., 2005; Darvasi and Shifman, 2005): the mestizo is valuable to both Brazil and Mexico as an object of genomic currency in the transnational enterprise of global medicine (Bustamante et al., 2011).

We have also warned against thinking of Latin American genomic research as homogeneous. The institutional activity of genomic research in Brazil is diverse, decentralized and detached from state agendas, while genomic research in Mexico is systematically saturated with state-supported interests.

Mixture as a process of national formation is not a singular ideology, but has been developed differently in different Latin American countries (Telles and Project on Ethnicity and Race in Latin America, 2014). Major features of Brazilian national identity include images of blackness, romanticized notions of the *índio*, and European whiteness, buttressed by old world mass migrations. Especially since the 1950s UNESCO studies, debates about racism and racial inequality – between whites, blacks and browns – have figured in discussions of Brazilian-ness. Brazil is also often used as a point of comparison in international discussions about race (Seigel, 2009). In Mexico, the primary category of otherness and potential assimilation is indigeneity, while blackness is a marginal category of otherness, and whiteness is only a minor locus of identity. Debates about race and racism in Mexico are minor, when compared with debates in Brazil.

There were also important differences between Brazil and Mexico in their respective political contexts for post-1990 multiculturalist reforms. A pioneer of constitutional and legal reform, Brazil has explicitly moved towards multiculturalism through its 1988 constitution, officially acknowledged racism as a significant problem in the 1990s, and rapidly adopted race-based affirmative action programmes. Mexico has been slower and less radical in these areas. Mexican measures to recognize indigenous communities – while offering departures from mid-century assimilationist *indigenismo* – have been criticized as vague and insubstantial, and the African contribution to the nation, *la tercera raíz* (the third root) has struggled to command a public space.

These differences have led to distinct Brazilian and Mexican mobilizations of the genomic mestizo. In Mexico, the state, geneticists and the medical community agree that improvements to health policy are the main goal of genomic research, despite disagreements over strategies and the roles of INMEGEN. And while multiculturalism and indigenous rights are certainly at issue in Mexico – witness the Zapatista movement – race is not a major controversy in genomic research. INMEGEN has had little to say openly about multiculturalism or race. As we have illustrated, the GM project implicitly reproduced the old divide between indigenous and mestizo identities, repackaging it in genetic idiom. This divide continues to guide approaches to national identity in post-multicultural reform Mexico, framing the genetic mestizo as paradigmatic. The presence of race is still significant, but implicit.

In contrast, in Brazil, race has been the main public arena of genomic research. Even debates about genomics and health – for example, in relation to provisions for sickle cell anaemia – have focused to some extent on the question of whether public health policies should differentiate by race (Maio and Monteiro, 2010). In such cases, questions of race have loomed larger than questions of health. What should the nation look like? Should racial difference be recognized and institutionalized in the public domain, or should the nation pursue a raceless society?

Our comparison of Mexico and Brazil shows the complex ways genetic data get entangled in debates about public policy, national identities and destinies. In many ways, GM and HB have been driven by common concerns in the field of global genetic medicine and population genetics; they have also been shaped by long-standing interests in mixture as a defining feature of the cultures and biological profiles of Latin American nations. Yet, the specific features of national histories, demographics and politics, as well as the ways states have decided to support genomic research, have resulted in different genomic mestizos in each country.

## Notes

1. This article is based on fieldwork carried out in Mexico by Vivette García-Deister, with the support of Carlos López-Beltrán, and in Brazil by Michael Kent, with the support of Ricardo Ventura Santos. García-Deister did ethnographic work in INMEGEN and interviewed scientists there and in other laboratories; Kent and Santos interviewed Sérgio Pena, while Kent also carried out interviews with other geneticists working in the field. These data are complemented by an analysis of scientific publications.

2. Cf. Hinterberger (2012b) on French Canada and M’charek et al. (2014b) on Europe, who also highlight regional differences beyond the United States.

3. Starting in the 1960s, there were also several research projects focusing on ‘isolated’ indigenous populations (Santos et al., 2014).

4. Pena has also worked in the areas of paternity testing, biomedicine, pharmacogenomics and indigenous populations, among others.

5. See http://www.youtube.com/watch?v=XuEOXwW6K88 and http://www.theguardian.com/commentisfree/2012/aug/14/brazil-free-dna-testing-rethink-race

6. The existence of a growing population of Mexicans living in the United States and being reethnicized as Latino certainly poses an intriguing series of questions for geneticists (Burchard et al., 2005; Montoya, 2011).

7. The website of Pena’s commercial laboratory offers to access to about 200 media items featuring Pena and his research, many of which are related to the debate on affirmative action (Gaspar Neto et al., 2012).

8. *Zacatecas@linea*, 23 September 2009, http://www.zacatecasonline.com.mx/noticias/local/739-genoma-zacatecas.html (accessed 21 January 2015); *El Universal*, 9 March 2007, http://www.eluniversal.com.mx/nacion/149089.html (accessed 21 January 2015).

## References

[bibr1-0306312715611262] Alcántara L (2007) Genes mexicanos, mezcla de 35 razas. El Universal, 9 3 Available at: http://archivo.eluniversal.com.mx/notas/411265.html

[bibr2-0306312715611262] Appelbaum NP Macpherson AS Rosenblatt KA (eds) (2003) Race and Nation in Modern Latin America. Chapel Hill, NC: University of North Carolina Press.

[bibr3-0306312715611262] Barahona A (2010) Historia de la Genética Humana en México, 1870–1970. México: Universidad Nacional Autónoma de México.

[bibr4-0306312715611262] Benjamin R (2009) A lab of their own: Genomic sovereignty as postcolonial science policy. Policy and Society 28(4): 341–355.

[bibr5-0306312715611262] Brodwin P (2002) Genetics, identity and the anthropology of essentialism. Anthropological Quarterly 75(2): 323–330.

[bibr6-0306312715611262] Burchard EG Borrell LN Choudhry S (2005) Latino populations: A unique opportunity for the study of race, genetics, and social environment in epidemiological research. American Journal of Public Health 95(12): 2161–2168. 16257940 10.2105/AJPH.2005.068668 PMC1449501

[bibr7-0306312715611262] Bustamante CD De La Vega FM Burchard EG (2011) Genomics for the world. Nature 475(7355): 163–165. 21753830 10.1038/475163a PMC3708540

[bibr8-0306312715611262] Cavalli-Sforza LL Menozzi P Piazza A (1994) The History and Geography of Human Genes. Princeton, NJ: Princeton University Press.

[bibr9-0306312715611262] Cunin E Hoffmann O (eds) (2013) Blackness and Mestizaje in Mexico and Central America. Trenton, NJ: Africa World Press.

[bibr10-0306312715611262] Daher A Jóia A Atila A (2008) 113 cidadãos anti-racistas contra as leis raciais. Available at: http://revistaepoca.globo.com/Revista/Epoca/0,,EDR83466-6014,00.html (accessed 3 October 2014).

[bibr11-0306312715611262] Darvasi A Shifman S (2005) The beauty of admixture. Nature Genetics 37(2): 118–119. 15678141 10.1038/ng0205-118

[bibr12-0306312715611262] De la Peña G (2006) A new Mexican nationalism? Indigenous rights, constitutional reform and the conflicting meanings of multiculturalism. Nations and Nationalism 12(2): 279–302.

[bibr13-0306312715611262] Freyre G (1936) Casa-Grande & Senzala; Formação da Familia Brasileira Sob o Regimen de Economia Patriarchal. Rio de Janeiro, Brazil: Schmidt.

[bibr14-0306312715611262] Fry PH (2005) A Persistência da Raça: Ensaios Antropológicos Sobre o Brasil e a África Austral. Rio de Janeiro, Brazil: Civilização Brasileira.

[bibr15-0306312715611262] Fry PH Maggie Y Monteiro S (eds) (2007) Divisões Perigosas: Políticas Raciais no Brasil Contemporâneo. Rio de Janeiro, Brazil: Civilização Brasileira.

[bibr16-0306312715611262] Fujimura JH Rajagopalan R (2011) Different differences: The use of ‘genetic ancestry’ versus race in biomedical human genetic research. Social Studies of Science 41(1): 5–30. 21553638 10.1177/0306312710379170 PMC3124377

[bibr17-0306312715611262] Fujimura JH Duster T Rajagopalan R (2008) Introduction: Race, genetics, and disease: Questions of evidence, matters of consequence. Social Studies of Science 38(5): 643–656. 19227816 10.1177/0306312708091926

[bibr18-0306312715611262] Fullwiley D (2008) The biologistical construction of race: ‘Admixture’ technology and the new genetic medicine. Social Studies of Science 38(5): 695–735. 19227818 10.1177/0306312708090796

[bibr19-0306312715611262] Gall O (ed.) (2007) Racismo, Mestizaje y Modernidad: Visiones desde Latitudes Diversas. Mexico: Universidad Nacional Autónoma de México.

[bibr20-0306312715611262] García-Deister V (2014) Laboratory life of the Mexican Mestizo. In: Wade P López-Beltrán C Restrepo E Santos RV (eds) Mestizo Genomics: Race Mixture, Nation, and Science in Latin America. Durham, NC: Duke University Press, 161–182.

[bibr21-0306312715611262] García-Deister V López-Beltrán C (2015) País de gordos/país de muertos: Obesity, death and nation in biomedical and forensic genetics in Mexico. Social Studies of Science 45(6): 797–815. 10.1177/0306312715608449 PMC4702212 27479997

[bibr22-0306312715611262] García Murcia MAA (2013) Profesionalización de la antropología física en México: la investigación, las instituciones y la enseñanza (1887–1942). Historia. México: Universidad Nacional Autónoma de México.

[bibr23-0306312715611262] Gaspar Neto VV Santos RV Kent M (2012) Biorrevelações: Testes de Ancestralidade Genética em Perspectiva Antropológica Comparada. In: Santos RV Gibbon S Beltrão JF (eds) Identidades Emergentes, Genética e Saúde: Perspectivas Antropológicas. Rio de Janeiro, Brazil: Editora Garamond & Editora Fiocruz, 233–267.

[bibr24-0306312715611262] Gledhill J (2002) The powers behind the masks: Mexico’s political class and social elites at the end of the millennium. In: Shore C Nugent S (eds) Elite Cultures. London: Routledge, 39–60.

[bibr25-0306312715611262] Gleizer D (2011) El Exilio Incómodo. México y los Refugiados Judíos, 1933–1945. México: El Colegio de México.

[bibr26-0306312715611262] *Globo* (2011) Genética derruba teses racialistas. Globo, 18 2, p. 7.

[bibr27-0306312715611262] Gómez Izquierdo JJ (1992) El Movimiento Antichino en México (1871–1934): Problemas del Racismo y del Nacionalismo Durante la Revolucion Mexicana. México: INAH.

[bibr28-0306312715611262] Gómez Izquierdo JJ Sánchez Díaz ME (2011) La Ideología Mestizante, el Guadalupanismo y sus Repercusiones Sociales. Una Revisión Crítica de la ‘Identidad Nacional’. Puebla: Universidad Iberoamericana de Puebla/Lupus Inquisitor.

[bibr29-0306312715611262] Guerrero Mothelet V Herrera S (2005) Mexico launches bold genome project. Nature Biotechnology 23(9): 1030. 10.1038/nbt0905-1030 16151379

[bibr30-0306312715611262] Guimarães AS (1999) Racismo e Anti-Racismo no Brasil. São Paulo, Brazil: Editora 34.

[bibr31-0306312715611262] Hartigan J (2013) Looking for race in the Mexican “Book of Life”: INMEGEN and the Mexican Genome Project. In: Hartigan J (ed.) Anthropology of Race: Genes, Biology, and Culture. Santa Fe, NM: School of Advanced Research Press, 125–150.

[bibr32-0306312715611262] Heath D Rapp R Taussig K-S (2004) Genetic citizenship. In: Nugent D Vincent J (eds) A Companion to the Anthropology of Politics. New York: Wiley-Blackwell, 152–167.

[bibr33-0306312715611262] Hinterberger A (2012a) Investing in life, investing in difference: Nations, populations and genomes. Theory, Culture & Society 29(3): 72–93.

[bibr34-0306312715611262] Hinterberger A (2012b) Publics and populations: The politics of ancestry and exchange in genome science. Science as Culture 21(4): 528–549.

[bibr35-0306312715611262] Hofbauer A (2006) Uma História do Branqueamento ou o Negro em Questão. São Paulo, Brazil: Editora UNESP.

[bibr36-0306312715611262] Hunt LM Truesdell N (2013) Observations on the tenacity of racial concepts in genetics research. In: Hartigan J (ed.) Anthropology of Race: Genes, Biology, and Culture. Santa Fe, NM: School for Advanced Research Press, 83–108.

[bibr37-0306312715611262] Jiménez-Sanchez G Childs B Valle D (2001) Human disease genes. Nature 409(6822): 853–855. 11237009 10.1038/35057050

[bibr38-0306312715611262] Jiménez-Sánchez G Frenk J Soberón G (2012) Design and implementation of a platform for genomic medicine in Mexico. In: Kumar D (ed.) Genomics and Health in the Developing World. Oxford: Oxford University Press, 1173–1191.

[bibr39-0306312715611262] Kaufmann RFM (2009) Arguição de Descumprimento de Preceito Fundamental 186–2/800. Brasilia, Brazil: Supremo Tribunal Federal.

[bibr40-0306312715611262] Kent M (2013) The importance of being Uros: Indigenous identity politics in the genomic age. Social Studies of Science 43(4): 534–556.

[bibr41-0306312715611262] Kent M Wade P (2015) Genetics against race: Science, politics and affirmative action in Brazil. Social Studies of Science 45(6): 816–838. 10.1177/0306312715610217 PMC4702207 27479998

[bibr42-0306312715611262] Koenig BA Lee SS-J Richardson SS (eds) (2008) Revisiting Race in a Genomic Age. New Brunswick, NJ: Rutgers University Press.

[bibr43-0306312715611262] Lewontin RC (1972) The apportionment of human diversity. Evolutionary Biology 6: 381–398.

[bibr44-0306312715611262] Lima NT Sá DM (eds) (2008) Antropologia Brasiliana: Ciência e Educação na Obra de Edgard Roquette-Pinto. Belo Horizonte, Brazil: Ed. UFMG.

[bibr45-0306312715611262] López-Beltrán C García-Deister V (2013) Scientific approaches to the Mexican mestizo. História, Ciências, Saúde – Manguinhos 20(2): 391–410. 10.1590/s0104-597020130002000002 23903910

[bibr46-0306312715611262] López-Beltrán C Vergara Silva F (2011) Genómica nacional: el INMEGEN y el genoma del mestizo. In: Beltrán CL (ed.) Genes (&) Mestizos: Genómica y Raza en la Biomedicina Mexicana. Mexico: Ficticia Editorial, 99–142.

[bibr47-0306312715611262] López-Beltrán C García-Deister V Rios Sandoval M (2014) Negotiating the Mexican mestizo: On the possibility of a national genomics. In: Wade P López-Beltrán C Restrepo E Santos RV (eds) Mestizo Genomics: Race Mixture, Nation, and Science in Latin America. Durham, NC: Duke University Press, 85–106.

[bibr48-0306312715611262] Magnoli D (2009) Uma Gota de Sangue: História do Pensamento Racial. São Paulo, Brazil: Editora Contexto.

[bibr49-0306312715611262] Maio MC (2001) UNESCO and the study of race relations in Brazil: Regional or national issue? Latin American Research Review 36(2): 118–136. 18087798

[bibr50-0306312715611262] Maio MC Monteiro S (2010) Política social com recorte racial no Brasil: O caso da saúde da população negra. In: Maio MC Santos RV (eds) Raça como Questão: História, Ciência e Identidades no Brasil. Rio de Janeiro, Brazil: Editora Fiocruz, 285–314.

[bibr51-0306312715611262] Maio MC Santos RV (2005) Política de cotas raciais, os “olhos da sociedade” e os usos da antropologia: O caso do vestibular da universidade de Brasília (UnB). Horizontes Antropológicos 11(23): 181–214.

[bibr52-0306312715611262] Montoya MJ (2007) Bioethnic conscription: Genes, race, and Mexicana/o ethnicity in diabetes research. Cultural Anthropology 22(1): 94–128.

[bibr53-0306312715611262] Montoya MJ (2011) Making the Mexican Diabetic: Race, Science, and the Genetics of Inequality. Berkeley, CA: University of California Press.

[bibr54-0306312715611262] M’charek A (2013) Beyond fact or fiction: On the materiality of race in practice. Cultural Anthropology 28(3): 420–442.

[bibr55-0306312715611262] M’charek A Schramm K Skinner D (2014a) Technologies of belonging: The absent presence of race in Europe. Science, Technology & Human Values 39(4): 459–467.

[bibr56-0306312715611262] M’charek A Schramm K Skinner D (2014b) Topologies of race: Doing territory, population and identity in Europe. Science, Technology & Human Values 39(4): 468–487.

[bibr57-0306312715611262] Nash C (2013) Genome geographies: Mapping national ancestry and diversity in human population genetics. Transactions of the Institute of British Geographers 38(2): 193–206.

[bibr58-0306312715611262] Navarrete Linares F (2009) La construcción histórica de la discriminación étnica. In: Di Castro E (ed.) Justicia, Desigualdad y Exclusión 3: México. México: Universidad Nacional Autónoma de México, 237–282.

[bibr59-0306312715611262] Nelson A (2008) The factness of diaspora: The social sources of genetic genealogy. In: Koenig BA Lee SS-J Richardson SS (eds) Revisiting Race in a Genomic Age. New Brunswick, NJ: Rutgers University Press, 253–268.

[bibr60-0306312715611262] Oliveira Vianna FJD (1923) Evolução do Povo Brasileiro. São Paulo, Brazil: Monteiro Lobato.

[bibr61-0306312715611262] Palmié S (2007) Genomics, divination and ‘racecraft’. American Ethnologist 34(2): 205–222.

[bibr62-0306312715611262] Pálsson G (2007) Anthropology and the New Genetics. Cambridge: Cambridge University Press.

[bibr63-0306312715611262] Pálsson G Rabinow P (1999) Iceland: The case of a national human genome project. Anthropology Today 15(5): 14–18. 15199942

[bibr64-0306312715611262] Parra FC Amado RC Lambertucci JR Rocha J Antunes CM Pena SD (2003) Color and genomic ancestry in Brazilians. Proceedings of the National Academy of Sciences of the United States of America 100(1): 177–182. 12509516 10.1073/pnas.0126614100 PMC140919

[bibr65-0306312715611262] Pena SDJ (ed.) (2002) Homo Brasilis: Aspectos Genéticos, Lingüísticos, Históricos e Socioantropológicos da Formação do Povo Brasileiro. Ribeirão Preto, Brazil: FUNPEC-RP.

[bibr66-0306312715611262] Pena SDJ (2005) Razões para banir o conceito de raça da medicina brasileira. História, Ciências, Saúde – Manguinhos 12(2): 321–346. 10.1590/s0104-59702005000200006 16323361

[bibr67-0306312715611262] Pena SDJ (2008) Humanidade Sem Raças? São Paulo, Brazil: Publifolha.

[bibr68-0306312715611262] Pena SDJ (2009) Igualmente Diferentes. Belo Horizonte, Brazil: Editora UFMG.

[bibr69-0306312715611262] Pena SDJ (2011) The fallacy of racial pharmacogenomics. Brazilian Journal of Medical Biology Research 44(4): 268–275. 10.1590/s0100-879x2011007500031 21412662

[bibr70-0306312715611262] Pena SDJ Birchal TS (2006) A inexistência biológica versus a existência social de raças humanas: Pode a ciência instruir o etos social? Revista USP 68: 10–21.

[bibr71-0306312715611262] Pena SDJ Bortolini MC (2004) Pode a genética definir quem deve se beneficiar das cotas universitárias e demais ações afirmativas? Estudos Avanzados 18(50): 31–50.

[bibr72-0306312715611262] Pena SDJ Bastos-Rodrigues L Pimenta JR Bydlowski SP (2009) DNA tests probe the genomic ancestry of Brazilians. Brazilian Journal of Medical and Biological Research 42(10): 870–876. 19738982 10.1590/s0100-879x2009005000026

[bibr73-0306312715611262] Pena SDJ Carvalho-Silva DR Alves-Silva J Prado VF Santos FR (2000) Retrato molecular do Brasil. Ciência Hoje 159: 16–25.

[bibr74-0306312715611262] Pena SDJ Di Pietro G Fuchshuber-Moraes M (2011) The genomic ancestry of individuals from different geographical regions of Brazil is more uniform than expected. PLoS ONE 6(2): e17063. 21359226 10.1371/journal.pone.0017063 PMC3040205

[bibr75-0306312715611262] Rabinow P (1996) Essays on the Anthropology of Reason. Princeton, NJ: Princeton University Press.

[bibr76-0306312715611262] Rabinow P (1999) French DNA: Trouble in Purgatory. Chicago, IL: University of Chicago Press.

[bibr77-0306312715611262] Reardon J (2005) Race to the Finish: Identity and Governance in an Age of Genomics. Princeton, NJ: Princeton University Press.

[bibr78-0306312715611262] Rose N Novas C (2005) Biological citizenship. In: Ong A Collier SJ (eds) Global Assemblages: Technology, Politics, and Ethics as Anthropological Problems. Oxford: Blackwell Publishing, 439–463.

[bibr79-0306312715611262] Rutsch M (2001) Ramón Mena y Manuel Gamio: Una mirada oblicua sobre la antropología mexicana en los años veinte del siglo pasado. Relaciones: Estudios de Historia y Sociedad 22(88): 79–118.

[bibr80-0306312715611262] Saade Granados M (2009) El Mestizo No Es ‘de Color’. Ciencia y Política Pública Mestizófilas (México, 1920–1940). México: Escuela Nacional de Antropología e Historia ENAH – INAH.

[bibr81-0306312715611262] Salzano FM Freire-Maia N (1970) Problems in Human Biology: A Study of Brazilian Populations. Detroit, MI: Wayne State University Press.

[bibr82-0306312715611262] Sanchez CA (2008) Heidegger in Mexico: Emilio Uranga’s ontological hermeneutics. Continental Philosophy Review 41(4): 441–460.

[bibr83-0306312715611262] Santos RV (2012) Guardian angel on a nation’s path: Contexts and trajectories of physical anthropology in Brazil in the late nineteenth and early twentieth centuries. Current Anthropology 53(S5): S17–S32.

[bibr84-0306312715611262] Santos RV Maio MC (2004) Race, genomics, identities and politics in contemporary Brazil. Critique of Anthropology 24(4): 347–378.

[bibr85-0306312715611262] Santos RV Fry PH Monteiro S (2009) Color, race and genomic ancestry in Brazil: Dialogues between anthropology and genetics. Current Anthropology 50(6): 787–819. 20614657 10.1086/644532

[bibr86-0306312715611262] Santos RV Lindee S de Souza VS (2014) Varieties of the primitive: Human biological diversity studies in Cold War Brazil (1962–1970). American Anthropologist 116(4): 723–735.

[bibr87-0306312715611262] Schwartz-Marín E (2011) Genomic Sovereignty and the Mexican Genome: An Ethnography of Postcolonial Biopolitics. PhD Dissertation, Sociology and Philosophy, University of Exeter, Exeter.

[bibr88-0306312715611262] Schwartz-Marín E Restrepo E (2013) Biocoloniality, governance, and the protection of ‘genetic identities’ in Mexico and Colombia. Sociology 47(5): 993–1010.

[bibr89-0306312715611262] Schwartz-Marín E Silva-Zolezzi I (2010) ‘The map of the Mexican’s genome’: Overlapping national identity, and population genomics. Identity in the Information Society 3(3): 489–514.

[bibr90-0306312715611262] Seguín B Hardy B-J Singer PA Daar AS (2008) Genomics, public health and developing countries: The case of the Mexican National Institute of Genomic Medicine (INMEGEN). Nature Reviews Genetics 9: S5–S9. 10.1038/nrg2442 18802419

[bibr91-0306312715611262] Seigel M (2009) Uneven Encounters: Making Race and Nation in Brazil and the United States. Durham, NC: Duke University Press.

[bibr92-0306312715611262] Sieder R (ed.) (2002) Multiculturalism in Latin America: Indigenous Rights, Diversity and Democracy. Basingstoke: Palgrave Macmillan.

[bibr93-0306312715611262] Silva-Zolezzi I Hidalgo-Miranda A Estrada-Gil J Carlos Fernandez-Lopez J Uribe-Figueroa L Contreras A (2009) Analysis of genomic diversity in Mexican Mestizo populations to develop genomic medicine in Mexico. Proceedings of the National Academy of Sciences of the United States of America 106(21): 8611–8616. 19433783 10.1073/pnas.0903045106 PMC2680428

[bibr94-0306312715611262] Simpson B (2000) Imagined genetic communities: Ethnicity and essentialism in the twenty-first century. Anthropology Today 16(3): 3–6.

[bibr95-0306312715611262] Souza VS de Santos RV (2014) The emergence of human population genetics and narratives about the formation of the Brazilian nation (1950–1960). Studies in History and Philosophy of Science Part C: Studies in History and Philosophy of Biological and Biomedical Sciences 47(Part A): 97–107. 10.1016/j.shpsc.2014.05.010 24954151

[bibr96-0306312715611262] Stepan NL (1991) ‘The Hour of Eugenics’: Race, Gender and Nation in Latin America. Ithaca, NY: Cornell University Press.

[bibr97-0306312715611262] Suárez-Díaz E (2014) Indigenous populations in Mexico: Medical anthropology in the work of Ruben Lisker in the 1960s. Studies in History and Philosophy of Science Part C: Studies in History and Philosophy of Biological and Biomedical Sciences 47(Part A): 108–117. 10.1016/j.shpsc.2014.05.011 25022488

[bibr98-0306312715611262] Suárez-Díaz E Barahona A (2013) Postwar and post-revolution: Medical genetics and social anthropology in Mexico (1945–1970). In: Gausemeier B Müller-Wille S Ramsden E (eds) Human Heredity in the Twentieth Century. London: Pickering and Chatto, 101–112.

[bibr99-0306312715611262] Suarez-Kurtz G Vargens DD Struchiner CJ Bastos-Rodrigues L Pena SD (2007) Self-reported skin color, genomic ancestry and the distribution of GST polymorphisms. Pharmacogenetics and Genomics 17(9): 765–771. 17700365 10.1097/FPC.0b013e3281c10e52

[bibr100-0306312715611262] TallBear K (2007) Narratives of race and indigeneity in the Genographic Project. Journal of Law, Medicine & Ethics 35(3): 412–424. 10.1111/j.1748-720X.2007.00164.x 17714251

[bibr101-0306312715611262] Taylor-Alexander S Schwartz-Marín E (2013) Bioprophecy and the politics of the present: Notes on the establishment of Mexico’s national genomics institute (INMEGEN). New Genetics and Society 32(4): 333–349.

[bibr102-0306312715611262] Telles EE (2004) Race in Another America: The Significance of Skin Color in Brazil. Princeton, NJ: Princeton University Press.

[bibr103-0306312715611262] Telles EE Project on Ethnicity and Race in Latin America (2014) Pigmentocracies: Ethnicity, Race and Color in Latin America. Chapel Hill, NC: University of North Carolina Press.

[bibr104-0306312715611262] Tenorio M (2006) Guatemala y México: Del Mestizaje a Contrapelo. Revista Istor 4(24): 67–94.

[bibr105-0306312715611262] Vasconcelos J (1997 [1925]). The Cosmic Race: A Bilingual Edition. Baltimore, MD: Johns Hopkins University Press.

[bibr106-0306312715611262] Vergara Silva F (2013) ‘Un asunto de sangre’: Juan Comas, el evolucionismo bio-info-molecularizado y las nuevas vidas de la ideología indigenista en México. In: Mansilla Lory J Lizárraga Cruchaga X (eds) Miradas Plurales al Fenómeno Humano. México: Instituto Nacional de Antropología e Historia, 235–265.

[bibr107-0306312715611262] Viqueira JP (2010) Reflexiones contra la noción histórica de mestizaje. Nexos, 76–83. Available at: http://www.nexos.com.mx/?p=13750

[bibr108-0306312715611262] Wade P (2010) Race and Ethnicity in Latin America. London: Pluto Press.

[bibr109-0306312715611262] Wade P García-Deister V Kent M Fernanda Olarte Sierra M Díaz Del Castillo Hernández A (2014a) Nation and the absent presence of race in Latin American genomics. Current Anthropology 55(4): 497–522.

[bibr110-0306312715611262] Wade P López-Beltrán C Restrepo E Santos RV (eds) (2014b) Mestizo Genomics: Race Mixture, Nation, and Science in Latin America. Durham, NC: Duke University Press.

[bibr111-0306312715611262] Wailoo K Nelson A Lee C (eds) (2012) Genetics and the Unsettled Past: The Collision of DNA, Race, and History. New Brunswick, NJ: Rutgers University Press.

